# Significantly Improved Electrical Properties of Photo-Initiated Auxiliary Crosslinking EPDM Used for Cable Termination

**DOI:** 10.3390/polym11122083

**Published:** 2019-12-13

**Authors:** Zhong-Yuan Li, Wei-Feng Sun, Hong Zhao

**Affiliations:** Key Laboratory of Engineering Dielectrics and Its Application, Ministry of Education, Heilongjiang Provincial Key Laboratory of Dielectric Engineering, School of Electrical and Electronic Engineering, Harbin University of Science and Technology, Harbin 150080, China; zli_ma16@hrbust.edu.cn

**Keywords:** electrical conductance, ultraviolet irradiation, crosslinking reaction, first-principles calculation, finite element simulation

## Abstract

In order to achieve high quality electrical materials for cable terminations, the crosslinked ethylene-propylene-diene monomer (EPDM) materials, with adequate breakdown strength, appropriately increased conductivity and are developed by employing auxiliary crosslinker and ultraviolet (UV) photoinitiated crosslinking technique. The characteristic cyclic anhydrides with coupled carbonyl groups are utilized as auxiliary crosslinkers to promote crosslinking efficiency and provide polar-groups to EPDM molecules in UV-initiated crosslinking processes, which can be effectively fulfilled in industrial cable production. The results of infrared spectroscopy show that the auxiliary crosslinkers have been successfully grated to EPDM molecules through UV initiation process. The conductivity of EPDM increases after individually utilizing three auxiliary crosslinkers to EPDM at various temperatures of cable operations, by which the highest conductivity has been acquired by grafting *N*.*N*-m-phenylene dimaleimide. The first-principles calculations demonstrate that some occupied local electronic-states have been introduced in the band-gap of the EPDM crosslinked by *N*.*N*-m-phenylene dimaleimide (EPDM-HAV2), which can be thermally excited from valence band to conduction band at lower temperature or in higher density, leading to augmentation in electrical conductivity. Meanwhile, the breakdown strength achieves a significant improvement in consistency with the theoretical estimation that deeper hole-traps can be introduced by auxiliary-crosslinking modification, and will consequently increase breakdown strength through the trapping mechanism of space charge suppression. in relation to the appropriately increased conductivity, in combination with persistent breakdown strength, the finite element simulations of the electric field distribution in EPDM cable terminations suggest that the effectively homogenized electric field at the root of stress cone will be realized for EPDM-HAV2. The present study offers a fundamental strategy to ameliorate EPDM materials in the application of insulated cable accessories.

## 1. Introduction

Ethylene propylene diene monomer (EPDM) is a representative terpolymer being copolymerized by ethylene, propylene, and non-conjugated diolefine. These belong to the polydiolefine family with the molecular backbone being completely saturated and only the branch containing unsaturated double bonds. The molecular structure characteristics of EPDM lead to high resistances to oxidation, chemical corrosion, and optical radiation, as well as the excellent vulcanization properties [1]. Among all the rubber, EPDM possesses the lowest specific gravity and can persist preferable electrical properties, even after absorbing a large number of fillers and oil. In particular, EPDM is essentially non-polar and not compatible with polar molecules, resulting in a considerably low rate of water absorption and the extraordinary insulating performances [2]. Although EPDM has been preferably used as a qualified insulation material in high voltage direct current (HVDC) cable accessories, there are still unaddressed technological issues for the interface between cross-linked polyethylene (XLPE) and reinforced insulating EPDM, primarily due to the discrepant electrical conductivity between XLPE and EPDM [3]. Accordingly, space charges are liable to accumulate at the interface between cables and insulation accessories under DC electric field, giving rise to severe local distortion of electric field in cable accessories, and eventually causing electrical breakdown [4,5]. Furthermore, converter transformer and other non-synchronous apparatuses will produce transient overloading in the operating HVDC cables, which can also exacerbate space charge accumulations in cable accessories. The flaws in electrical conductivity of dielectric materials, that could not be pertinently utilized for cable accessories, are now substantially restricting the development of HVDC transmission [6].

Since the nano-dielectrics (polymer dielectric nanocomposites) were proposed in the 1990s, the underlying modification mechanism and realizing their applications in industrial production of insulated cables are always the most important issues. It is found that the dielectric properties of polymers can be significantly amended by filling nanoparticles. In order to investigate novel insulation materials to homogenize the electric field in cable accessories, the nonlinear nanocomposites have been focused as prospective candidates by which the electrical conductivity or dielectric permittivity will suddenly increase with the increment of applied electric field strength, due to back-to-back Schottky barrier formed by the accumulated charges at nano-interfaces [7,8,9,10]. The insulating materials with nonlinear dielectric properties can homogenize electric field distribution and thus reduce space charge accumulation caused by electric field distortion. Non-linear dielectric materials can overcome the technical problems that cannot be solved in traditional fabrication processes and structure design of cable manufactures. The nonlinear conductivity of nanocomposites depends on the effective contact area between nanoparticles and polymer matrix, which requires the nanofiller to be controlled with respect to the minimized size and highly dispersed distribution [11]. Meanwhile, in order to obtain nonlinear composites, the high content of non-linear nanofillers to achieve nonlinear conductivity will cause the deterioration of mechanical properties and breakdown strength. Therefore, these drawbacks and difficulties in developing cable accessories by nanodielectrics technology make it almost impossible to be fulfilled in practical industrial productions. Novel modification strategies are urgently needed to circumvent the inevitable limitations of nanodielectrics. At present, the other effective methods of modifying polymer insulation materials include ultra-clean process, blending, and chemical modification [12]. Based on the first-principles calculations, the energetic features of intrinsic traps, introduced by physical and chemical defects, have been reported to reasonably suggest that the polar group can present deep traps in polymer materials [13,14]. Recent reports indicated that the excellent dielectric properties of modified polymers via chemical grafting are attributed to the trapping mechanism of space charge suppression and breakdown strength improvement [15,16]. However, due to low gasification temperature, the grafted molecules are liable to vaporize in chemical grafting reactions to form gas bubbles in the pipeline of polymer material production, which will intensively deteriorate the insulation performance. It is inevitable for chemical grafting reactions to produce by-product impurity, which need to be carried out at high temperature and pressure for a long time, leading to severe mechanical degradation of polymer materials. Therefore, the traditional chemical method of grafting micro-molecules cannot be applied for industrial production of cable accessories.

In the present study, in order to fulfill the prospective molecular modification of polymer insulation materials, a new tactical scheme of initiating crosslinking reactions of the EPDM and the auxiliary crosslinking agents with polar-group by ultraviolet (UV) irradiation is employed. This results in the crosslinked EPDM, with preferable dielectric properties, to be specifically utilized in industrial productions of cable terminations. Accordingly, we adopt *N*.*N*-m-phenylene dimaleimide (HVA2), triallyl isocyanurate (TAIC) and trimethylolpropane trimethacrylate (TMPTMA) as auxiliary crosslinking agents, and benzophenone (BP) as photoinitiator to carry out crosslinking reactions under UV irradiation. Theoretically, the first-principles electronic structure calculations indicate that electronic bound states with the energy level located in the band-gap of EPDM arise after EPDM molecules are connected by auxiliary crosslinkers, which account for the underlying mechanism of increasing conductivity and breakdown strength. The electric field distributions in cable terminations are constructed with modified crosslinked EPDM, and are finally investigated by finite element numerical simulations.

## 2. Experiments, Theoretical Calculations, and Finite Element simulations

### 2.1. Material Synthesis

The melting blend and hot-pressing approaches are employed through the synthesis of modified EPDM with the basic raw materials being presented as follows: EPDM (4725P, American DuPont Co., Ltd., Chicago, Illinois, USA) as parent material, benzophenone (BP, Jinleiyuan Chemical Co., Ltd., Lianyungang, China) for initiating grafting reaction under photon irradiation, auxiliary crosslinking agents (HVA2, TAIC and TMPTMA, Sinopharm Chemical Reagent Co., Ltd., Shanghai, China), all of which are in the purity higher than 95%. In the melting blend process of preparing the initial mixtures, the pristine EPDM is melted uniformly in Torque Rheometer (RM200C, Hapro Company Ltd., Harbin, China) at 140 °C for 11 min with a stirring speed of 60 rpm, and then 2 wt % BP photon-initiator and 1 wt % auxiliary crosslinking agent are added, then blended for 4min and cooled down to room temperature so as to obtain the uniform mixture materials, which will eventually be pressed into film specimens under a pressure of 15 MPa for 30 min at 140 °C. For photon-initiated crosslinking reactions, the prepared hot-pressed blend is firstly treated in a flat vulcanizer at 120 °C with the pressure being increased by 5 MPa per 5 min from 0 to 15 MPa so as to make the material melt, and then the melt material is irradiated by a light source array of UV LED units (NVSU233A-U365, Riya Electronics Chemistry Co., Ltd., Shanghai, China) for 2 s on a pre-irradiation platform at the normal pressure and room temperature in air atmosphere. The electrically modified EPDM materials are finally achieved after short-circuit degassing at 80 °C for 48 h in a vacuum oven so as to eliminate the residual impurities of small molecules. In photon-initiated crosslinking process under UV irradiation, the power and wavelength of light-emitting are controlled on 1.0 W, and 365 nm, respectively, and light incident direction is 60° angle with the plane of thin film sample. Besides, in order to ensure homogeneous crosslinking reactions across the film plane of EPDM materials, the samples are mounted on the conveyor belt at a constant speed of 1.5 mm/s with a distance of 15 mm between the film plane and UV light source.

### 2.2. Characterization and Measurement

In order to verify whether the auxiliary crosslinkers are grafted onto EPDM molecular chains through UV-initiated crosslinking reactions, the molecular structures of prepared homologous composites and crosslinked EPDM are characterized by Fourier Transform Infrared (FT-IR) Spectroscopy (FT/IR-6100, Jiasco Trading Co., Ltd., Shenyang, China) in the spectral range of 500–4000 cm^−1^ with the scanning resolution of 2 cm^−1^. In accordance to the standards of GB/T 2951.21-2008, and ASTM D 2765-2011, respectively, the crosslinking degrees of EPDM are verified through thermal elongation and gel extraction experiments in which the prepared materials are pressed into dumbbell-shaped samples under 0.2 MPa and then degassed in a vacuum oven at 200 °C for 30 min.

Electrical conductivity is tested by a three-electrode system at various temperatures from 30 to 70 °C for the circular film samples of 50 mm diameter and 300 µm thickness with the evaporated aluminum electrodes on both sides. The protective electrode in annular shape (inner and outer diameters of 54, and 76 mm, respectively) encircles the disc of measuring electrode (50 mm in diameter) on one side of the film samples, and the circular electrode with a larger diameter of 78 mm on the other side is used for applying high voltage. The three-electrode system is composed of a high-voltage DC power supply (continuously adjustable output voltage from 0 to 15 kV), an Ammeter with a testing range of 10^−4^–10^−15^ A) and an oven with a maximum operating temperature of 200 °C. After the tested samples are preheated in the oven for 1 h with the protection, high voltage and measuring electrodes being connected to ground, DC power supply, and Ammeter, respectively, DC power voltage is increased gradually to each level of testing voltage (electric field covering the range of 3–40 kV/mm) keeping for 1 h, on which the stable conductance current and voltage values are recorded. In order to reduce random error in testing experiments, three identical samples are prepared for each testing point of electrical conductance under the same condition, by which the averaged values are obtained as the final results to be plotted into conductivity-electric field curves. The DC dielectric breakdown strength of the circular film samples with a diameter of 80 mm and a thickness of 0.1 mm are tested with asymmetric columnar electrodes (25 and 75 mm in diameter for high voltage, and ground electrodes, respectively) by recording the maximum voltage before the sample breakdown when the applied electric field is raised continuously at a constant speed of 4 kV/s.

### 2.3. Molecular Model and Theoretical Methodology

The molecular models of EPDM molecules are chemically connected by auxiliary crosslinker and initially constructed with random distributed torsion, by which the EPDM molecules of 20 polymerization degree are crosslinked by auxiliary crosslinker molecule near the middle position of EPDM backbone chain, based on rotational isomeric state (RIS) model [17]. The constructed initial polymer configurations are geometrically optimized to structural relaxation by total energy functional minimization with conjugated gradient algorithm in first-principles calculations [18], so that the energy change, atomic force and displacement are theoretically evaluated to be lower than 1.0 × 10^−5^ eV/atom, 0.03 eV/Å, and 0.001 Å, respectively. The electronic structures are calculated based on the molecular orbitals and electronic density of states to investigate the band-edge features and grafting-introduced trap states. The first-principles calculations are performed by employing the scheme of all-electron and numerical atom-orbitals as implemented in DMol3 program of Materials studio 8.0 software package (Accelrys Inc., Materials Stutio v8.0.0.843, San Diego, CA, USA), as the detailed methodology adopted in calculations listed in Table 1.

### 2.4. Finite Element Simulations

**Figure 1:**
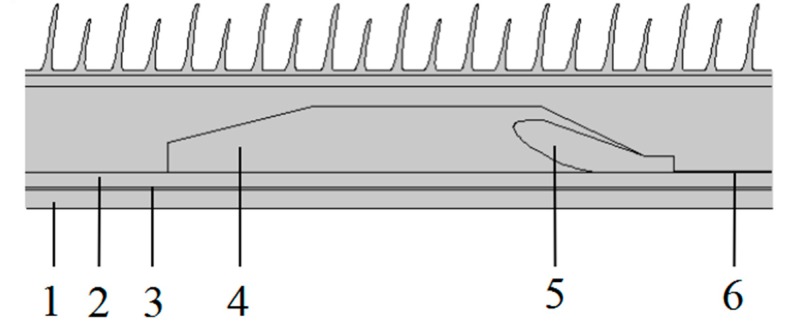
Schematic cable termination model: 1-core; 2-cross-linked polyethylene (XLPE); 3-inner shield; 4-reinforced insulation; 5-stress cone; 6-outer shield.

In order to investigate the electric field homogenization, caused by appropriately increasing the conductance in the reinforced insulation layer of modified EPDM, the HVDC cable termination under a voltage level of 200kV is simulated by finite element numerical schemes of electric-thermal field coupling [20] for the structure schematically shown in Figure 1. The cable termination is modeled by the geometry as follows: Diameter of core is 38mm, and the thicknesses of XLPE insulation, inner shield, outer shield and reinforced insulation are 16, 2, 1, and 68 mm, respectively. In our simulations, the core and ambient temperatures are set at 70 and 20 °C, with the electrical and thermal parameters of each constituting part, as listed in Table 2, substantially approaching the actual cable termination [21]. As implemented in the multi-physics field coupling module of COMSOL finite-element software package, the electric field distributions in cable termination are particularly evaluated, which are self-consistently coupling with thermal field. In the finite element simulations with COMSOL, the free triangular mesh generation is adopted to refine local mesh at the position where electric field changes greatly in cable termination. According to Delaunay triangulation algorithm, the model is divided into 211593 elements in total with the maximum and minimum elements are adjusted until the obtuse-angle triangulation disappears. The element growth rate is set to 1.5 and the relaxation degree of narrow region is set to 1 in mesh generation. The chosen mesh allows the finite-element solutions to be obtained independent of element size.

## 3. Results and Discussion

### 3.1. Material Characterization

Thermal elongation and gel extraction experiments are carried out by referring to the standards of GB/T 2951.21-2008, and ASTMD 2765-2011, respectively. The results for all the samples are listed in Table 3. It appears that the EPDM crosslinking degrees being initiated by UV-irradiation with various auxiliary crosslinking agents have reached an adequately high level to be mechanically qualified for cable terminations. This result proves the competent efficiency of UV-initiation crosslinking technique with auxiliary crosslinking agents.

**Figure 2:**
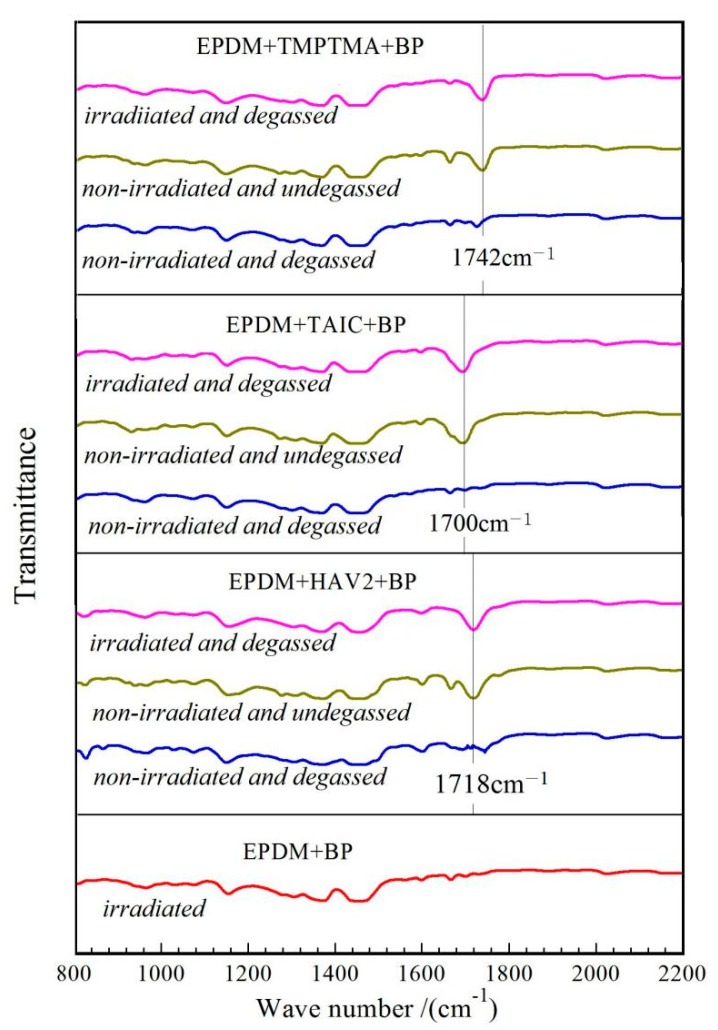
Infrared transmission spectra of ethylene-propylene-diene monomer (EPDM) + Auxiliary + benzophenone (BP) mixture in UV-initiation process (Auxiliary = TMPTMA, TAIC or HAV2), together with EPDM + BP mixture in comparison.

Auxiliary crosslinkers grafted on EPDM molecular chains are characterized by IR spectroscopy in comparison to the homologous blend sample obtained though the similar preparation process without UV irradiation or degassing treatment, as the results shown in Figure 2, implying the molecular structural changes caused by UV initiated crosslinking reactions. The TMPTMA, TAIC and HAV2 can be identified by the stretching vibration peaks of carbonyl (C=O) at 1742, 1700, and 1718 cm^−1^ respectively in IR transmission spectra, which are engendered by UV irradiation and are retained after hot-degassing treatment. Conversely, for the mixture of EPDM and BP without auxiliary crosslinking agents, these characteristic peaks are not presented even through the UV irradiation process, which confirms the definite correlation of these characteristic peaks to the auxiliary crosslinkers being chemically connected (grafted) between EPDM molecules. The FT-IR results reasonably demonstrate that the auxiliary crosslinking agents have been successfully grafted to EPDM molecules.

### 3.2. Electrical Conductance and Dielectric Breakdown Strength

**Figure 3:**
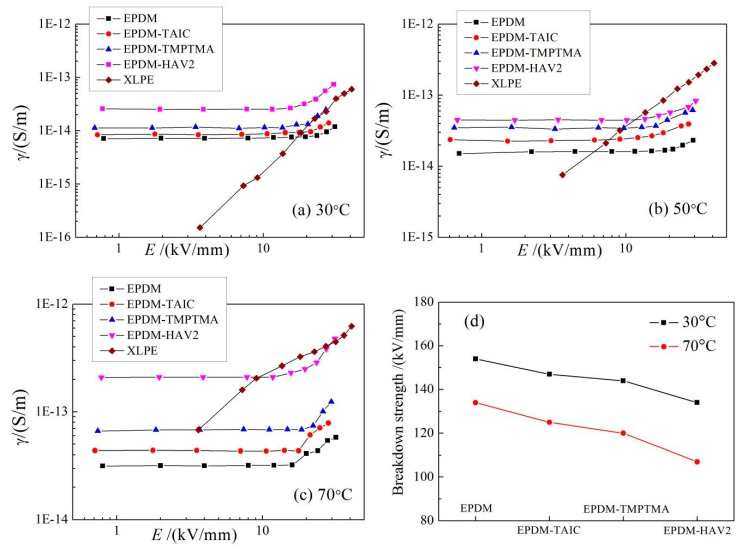
(**a**–**c**) *γ*-*E* curves and (**d**) characteristic dielectric breakdown strength (DBS) of EPDM materials at 30–70 °C. The conductivity property of XLPE also being presented for analyzing electric field distribution in cable termination.

The electrical conductance of EPDM materials is highly dependent on temperature and electric field, which is explicitly verified by the conductivity-electric field (*γ*-*E*) varying curves at the archetypal operating temperature of DC insulated cable (30–70 °C), as shown in Figure 3a–c. The conductivities of modified EPDM materials are appreciably higher than pure EPDM with the largest improvement being acquired by EPDM-g-HAV2. Moreover, based on the gradients of *γ*-*E* curves, the electrical conductance can be distinguished into Ohm region and trap active region, where the demarcation point represents the critical electric field that the dominant carrier transport alters from Ohm conductance to charge trap scattering [22]. The *γ*-*E* curves in Figure 3 also indicate the evident increment of critical electric field caused by grafting auxiliary crosslinking agents onto EPDM molecules. The two-parameter Weibull statistics are utilized to analyze the experimental data, as the fitted results of dielectric breakdown strength (DBS) shown in Figure 3d. The DBS of UV-initiated auxiliary-crosslinking EPDM is slightly lower than that of pure EPDM with the most reduced characteristic DBS approaching to 104 kV/mm at 70 °C for EPDM-HAV2, which preserves sufficient suitability for cable accessories [23].

**Figure 4:**
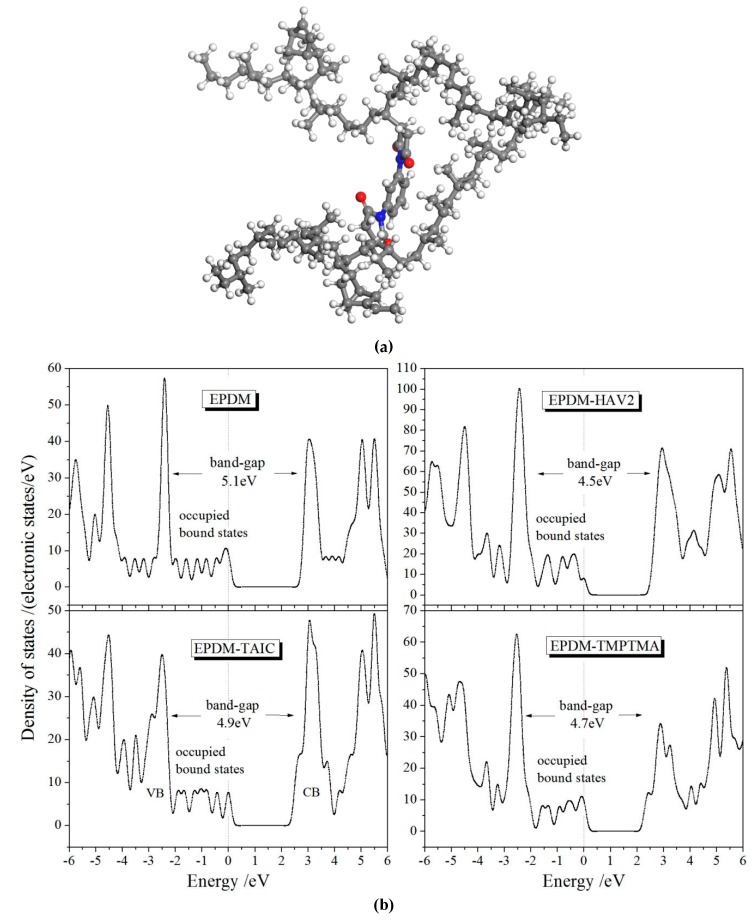
(**a**) Schematic molecular structure of EPDM-HAV2,( **b**) Density of states for EPDM molecules being chemically connected by different auxiliary crosslinkers in which the highest occupied molecular orbital (HOMO) is referenced as energy zero level indicated by vertical dashed lines.

In order to render the theoretical basis for the experimental results of conductivity and DBS, the first-principles electronic structure calculations are performed for crosslinked EPDM models as a multiple of EPDM molecules being chemically connected by an auxiliary crosslinker. The representative structural schematics of relaxed EPDM-HAV2 molecule and the energetic distribution of electronic states (density of states, DOS) are shown in Figure 4. The density of states near conduction band minimum (CBM) and valance band maximum (VBM) of polymer molecules pertains to the probability of the carrier transition between the adjacent levels of electronic states at band-edge, which thus dominate the carrier mobility without consideration of impurity or defect scattering. Accordingly, as illustrated by Figure 4b in comparison, plenty of occupied electronic states have been introduced by grafting HVA2, resulting in substantially higher DOS in both conduction and valance bands, which means the considerably higher carrier mobility has been acquired, and thus, consistently explains the highest conductivity of EPDM-HAV2 as the experimental results of Figure 3a–c. According to electronic structure theory of condensed matter physics, carrier mobility can be reduced from carrier-phonon scattering only when the temperature being highly raised, for which we can eliminate the temperature effect on carrier mobility of EPDM materials at 30–70 °C. Furthermore, the thermal excitation of electrons through reduced transition energy will cause increment of carrier density at band-edge, the probability of which also increases with increasing temperature in near ambient range for the transition energy values of EPDM materials. Therefore, the conductivity of EPDM molecules being crosslinked by an auxiliary crosslinker is evidently promoted as the temperature is raised in the cable working range of 30–70 °C. It is also observable for all the auxiliary-crosslinked EPDM from Figure 4b that multiple occupied electronic states can been introduced by grafting auxiliary crosslinker with the energy levels higher than the highest occupied molecular orbital (HOMO) of pure EPDM. This leads to a reduced transition energy from HOMO to lowest unoccupied molecular orbital (LUMO), which produces a higher carrier density at the same temperature. These theoretical results elucidate the underlying molecular physics of improving electrical conductivity consistently with the experimental EPDM modification of grafting auxiliary crosslinking agents by way of UV-initiated crosslinking process.

### 3.3. Electrical Field Distribution in DC Cable Termination

Due to the presence of temperature and electric field gradients that are generated in HVDC cable during steady state operation, conductivity perturbations may lead to local distortions of electric field and eventually cause electrical breakdown. Accordingly, the electric field distortion derives essentially from the conductivity discrepancy between cable insulation and reinforced insulation with XLPE, and EPDM as dielectric materials, respectively, especially for high cable operating temperatures. The electrical conductivity of XLPE increases intensively with the rises of temperature and electric field, which are attributed to thermal electronic-excitation, and impact ionization, respectively. When the temperature increases from 30 to 70 °C, the electrical conductivity of XLPE will go up by three and one orders of magnitude under low, and high fields, respectively, as compared in Figure 3a–c. Hence for cable termination, it is difficult to achieve sufficiently high conductivity approaching that of XLPE, while persisting an adequate insulation strength under typical cable working conditions, as the contrasting results shown in Figure 3, implying that the conductivities of all samples except EPDM-HAV2 are lower than that of XLPE. Prominently, almost identical conductivity has been acquired by EPDM-HAV2 in a high field region at a representative cable operating temperature of 70 °C, while retaining adequately high DBS. Therefore, it is reasonably suggested that the EPDM-HAV2 will be capable of restraining electric field distortion and contribute to homogenization.

**Figure 5:**
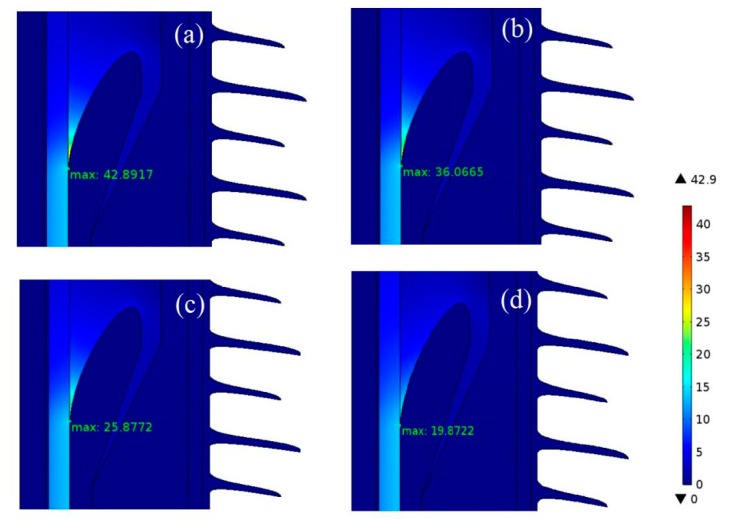
Simulated electric field distribution in cable terminations being constructed with (**a**) EPDM, (**b**) EPDM-TAIC, (**c**) EPDM-TMPTMA, and (**d**) EPDM-HAV2 as the dielectric materials in reinforced insulation layer under 200 kV/mm at the cable core temperature of 70 °C.

In the interest of analyzing the ability of modified EPDM materials for homogenizing the electric field in cable termination, the COMSOL multi-physics finite element simulations represent the distribution characteristics of electrostatic electrical fields, with the voltage and temperature of core regions being set as 200 kV, and 70 °C, respectively, as in Figure 5. It has been attested that the maximum electric field in the HVDC cable termination locates in the reinforced insulation layer near the root of stress cone, approaching to 43, 36, 25, and 20 kV/mm for pure EPDM, EPDM-TAIC, EPDM-TMPTMA, and EPDM-HAV2, respectively. This confirms the ability of EPDM-HAV2 to ameliorate the distribution of electric fields in reinforced insulation layer.

## 4. Conclusions

Employing auxiliary crosslinking agents, a specific crosslinked EPDM material with significantly increased conductivity and adequate insulation strength has been developed by UV-initiation crosslinking technique. The underlying mechanism of promoting electrical conductance are elucidated by quantum mechanics calculations in combination with conductivity-electric field variation curves, at various temperatures of operating insulated cable. The electrical compatibility of modified EPDM materials is proven by finite element numerical simulations of electric field distributions, in order to avoid electric field distortion in cable termination. It has been demonstrated that UV cross-linking technique can successfully initiate crosslinking reactions of EPDM molecules and auxiliary crosslinking agents to achieve modified crosslinked EPDM materials with sufficient crosslinking degree and excellent thermal elongation performance. Compared with pure EPDM, higher conductivity has been acquired by the modified EPDM materials with auxiliary crosslinkers, by which HVA2 represents prominent efficiency for ameliorating electrical performances. The first-principles calculations show that multiple electronic states have been engendered by the grafted auxiliary crosslinkers which will reduce band-gap and increase the density of state, in both conduction and valance bands, consequently leading to increments of carrier mobility and density. Finite element numerical simulations verify that EPDM-HAV2 can most effectively homogenize electric field distribution at the root of cable termination.

## Figures and Tables

**Table 1:** Schemes and parameters adopted in the first-principles calculations by DMol3.

Electronic Hamiltonian	Scheme	Condition and Parameter
Exchange-correlation energy	Meta-generalized-gradient approximation	M11-L [19]
Integration accuracy		2000 grid points /atom
SCF	Tolerance	1 × 10^−6^ eV/atom
	Multipolar expansion	Octupole
	Charge density mixing	Charge = 0.3, DIIS = 5
Core treatment	All Electron	
Numerical basis set	DNP	Basis file 4.4
Orbital cutoff	Global	5.0 Å

**Table 2:** The electrical and thermal parameters of materials adopted in electric field simulations.

Materials	Density (g/cm^3^)	Relative Permittivity	Specific Heat Capacity (J/kg·K)	Coefficient of Heat Conductivity
XLPE	910	2.27	1640	0.285
inner shield	950	100	2500	0.291

**Table 3:** Thermal extensibility and gel content for the crosslinked ethylene-propylene-diene monomer (EPDM) with various auxiliary crosslinkers.

Samples	Thermal Extensibility /%	Gel Content /%
EPDM-TMPTMA	25	87
EPDM-TAIC	25	85
EPDM-HAV2	20	92
EPDM+DCP	35	84
